# BiTUGA: scalable prevalence-based unitig association testing for binary traits

**DOI:** 10.1093/g3journal/jkag149

**Published:** 2026-06-15

**Authors:** Jannes Mittelbach, Birgit Kersten, Stefan Kurtz

**Affiliations:** Center for Bioinformatics, MIN-Faculty, University of Hamburg, 22761 Hamburg, Hamburg, Germany; Genome Research, Thünen Institute of Forest Genetics, 22927 Großhansdorf, Schleswig-Holstein, Germany; Center for Bioinformatics, MIN-Faculty, University of Hamburg, 22761 Hamburg, Hamburg, Germany

**Keywords:** alignment-free, *k*-mers, sex determination, genome-wide association studies, plant genomics

## Abstract

Sequence-based association studies can be challenging in large and highly repetitive genomes. While reference-free *k*-mer approaches enable direct analysis of sequence variation from sequencing reads, current tools often rely on constructing full *k*-mer-by-sample matrices, creating computational bottlenecks. Here, we present BiTUGA, a pipeline to test associations for discrete binary traits designed to overcome these limitations and optimized for large genomes. Shifting the statistical focus from raw abundance to group-level prevalence, BiTUGA assembles filtered *k*-mers into unitigs and tests unitig presence for association across groups. We validated BiTUGA on the binary trait sex, targeting structurally complex Sex-Determining Regions (SDRs) that remain largely unresolved in many plant species. BiTUGA identified sex-associated unitigs, detecting the known SDRs in *Populus tremula* and *Ginkgo biloba*, processing up to 750 Gbp in 14–25 h within 70 GB RAM. BiTUGA is available at https://github.com/JMittelbach/BiTUGA.git.

## Introduction

Genome-wide association studies (GWASs) based on single-nucleotide polymorphisms (SNPs) link genetic variation to phenotypic traits by mapping reads to a linear reference genome sequence (Visscher et al. 2017). In plants, large, repetitive, heterozygous, or polyploid genomes and structurally variable loci can complicate association analyses (Kyriakidou et al. 2018; Vicient and Casacuberta 2017; Garg et al. 2024; Kong et al. 2023). Reference-free *k*-mer-based approaches can complement SNP-based analyses by capturing signals linked to SNPs, indels, copy-number variation, and structural variants without requiring read mapping to a reference genome (Karikari et al. 2023).

Reference-free strategies operate directly on *k*-mers, substrings of fixed length *k* extracted from reads (Rahman et al. 2018). These methods treat each distinct *k*-mer as a possible marker, enabling variant detection in genomic regions that may be difficult to represent in a single reference genome sequence. In plants, *k*-mer-based GWAS has identified associations linked to copy number variation (CNV), introgression, and novel insertions invisible to standard SNP callers (He et al. 2024; Voichek and Weigel 2020).

However, applying *k*-mer methods to large genomes introduces computational challenges. In multi-gigabase sequencing datasets the number of distinct *k*-mers can exceed billions (Liao et al. 2020; Nystedt et al. 2013). Approaches constructing full *k*-mer-by-sample matrices, such as HAWK (Rahman et al. 2018), require aggregating counts across the entire sample set simultaneously. Constructing global matrices at this scale is computationally demanding. For discrete binary traits, where the dominant signal is expected to be a strong between-group presence/absence contrast, the cost of constructing dataset-wide abundance matrices is often disproportionate to the additional information gained over prevalence-based representations. The filtering tool KmerGO (Wang et al. 2020) targets group-specific sequences, but by indexing the entire *k*-mer spectrum, it may encounter scalability limits for large datasets (Holley and Melsted 2020; Marchet et al. 2021). Differential-counting tools like kmdiff (Lemane et al. 2022) offer efficiency but rely on abundance differences. Abundance-based methods may be confounded by repeats or CNVs (Benjamini and Speed 2012; Love et al. 2014), while treating *k*-mers as independent entities limits genomic context and biological interpretability (Jaillard et al. 2018). Graph-based approaches like DBGWAS (Jaillard et al. 2018) and MUSET (Vicedomini et al. 2025) compact *k*-mers into unitigs, but global graph construction remains memory-intensive (Chikhi and Rizk 2013; Holley and Melsted 2020).

These limitations are particularly acute in the study of dioecious plants, where sex is governed by sex-determining regions (SDRs) (Charlesworth 2016; Leite Montalvão et al. 2021). SDR identification is often obscured by huge, repetitive genomes, typical of many gymnosperms (Walas et al. 2018; Wan et al. 2022). Previous studies utilized strict presence/absence approaches to identify sex-specific *k*-mers, offering a resource-efficient alternative to complex association approaches (Akagi et al. 2014; Carey et al. 2024). However, heterogeneous sequencing depth and phenotypic misclassification make perfect segregation unreliable (Benjamini and Speed 2012). Therefore, robust identification requires group prevalence rather than strict intersection.

Here, we present BiTUGA (**Bi**nary **T**rait **U**nitig **G**enome-wide **A**ssociation), a scalable pipeline for reference-independent detection of genomic sequences associated with binary traits. BiTUGA implements a prevalence-based strategy avoiding global *k*-mer matrices. Robust to sequencing depth variation, it enables reproducible trait-associated sequence discovery.

## Methods

### Overview

**Fig. 1.**
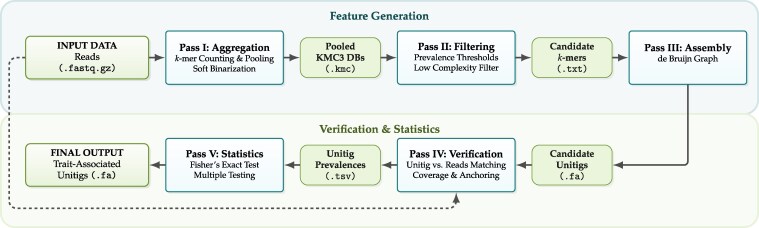
Sequential workflow of BiTUGA. **Feature Generation:** Reads are processed to generate features (unique *k*-mers) through *k*-mer counting, pooling, and soft binarization (Pass I), filtered by prevalence and complexity (Pass II), and assembled into unitigs (Pass III). **Verification & Statistics:** Unitig group prevalence is assessed by mapping unitigs back to input reads of each sample (Pass IV). Finally, trait-associated sequences are identified with Fisher’s Exact Test and optional multiple-testing correction (Pass V).

BiTUGA operates directly on Whole Genome Sequencing (WGS) reads. The tool accepts single-end and paired-end FASTQ files. Parameters are optimized for short-reads and are configurable to the number of input reads. BiTUGA proceeds in five passes (Fig. 1). The final output consists of significantly associated unitigs in FASTA format, with summary statistics and quality control plots. Outside of the core workflow of BiTUGA, these unitigs can be mapped to a reference assembly, if available. Here, the term *feature* refers to a unique *k*-mer from the input read set.

### Aggregation of *k*-mers and prevalence encoding (Pass I)

All canonical *k*-mers (default: k=31) are counted using KMC3 (Kokot et al. 2017). This *k*-mer length is consistent with standard reference-free association workflows (Rahman et al. 2018; Voichek and Weigel 2020). We convert the *k*-mer counts into sample prevalence using a soft-binarization strategy. Let $R_{s}$ denote the set of reads for a given sample *s*. For every canonical *k*-mer *w* observed in $R_{s}$, let $a_{s}(w)$ be its abundance count. To filter sequencing noise, for each sample *s*, singletons ($a_{s}(w)=1$) are discarded. There are no constraints on the maximum number of occurrences, to capture high-abundance *k*-mers.

To minimize disk usage, BiTUGA processes samples sequentially, using kmc to construct each sample database $D_{s}$ from $R_{s}$. After merging $D_{s}$ into its trait-specific pool using kmc_tools union, the individual database is deleted. Consequently, disk usage during this pass is bounded by the largest per-sample database and the two trait-specific pools. For a trait group *T* with a set of samples $S_{T}$, the aggregated group count $C_{T}(w)$ for *k*-mer *w* is the number of samples $s\in S_{T}$ such that $a_{s}(w)\geq 2$.

### Feature reduction and streaming filters (Pass II)

BiTUGA streams aggregated databases via the KMC3 API to identify candidate *k*-mers. We apply a prevalence filter to remove noise and uninformative core genomic regions. Let *n* be the total number of samples and C(w) be the number of samples containing *k*-mer *w*. For given parameters $\tau_{\min}$ and $\tau_{\max}$, a *k*-mer *w* is retained if


(1) $$\tau_{\min}\leq \frac{C(w)}{n}\leq \tau_{\max}.$$

To address class imbalance, thresholds are determined adaptively using the minority group ratio $r_{\min}$ and a penetrance factor *γ* (default: 0.5). For trait groups of equal size, we have $r_{\min}=0.5$, and thus $\tau_{\min}=0.25$ and $\tau_{\max}=0.75$. This approach acts as a trait-agnostic filter, preserving statistical neutrality in the workflow.

To reduce the number of *k*-mers even further, an optional filtering mode using the --group-contrast option can be applied. Let $P_{\mathrm{target}}(w)$ and $P_{\mathrm{opp}}(w)$ denote the prevalences (sample count divided by group size) for *k*-mer *w* in the target group and the opposing group. This heuristic retains *k*-mers satisfying


(2) $$P_{\mathrm{target}}(w)\geq \phi_{\min}\;\text{and}\;P_{\mathrm{opp}}(w)\leq \phi_{\max},$$

with $\phi_{\min}=0.50$ and $\phi_{\max}=0.30$ by default. This enforces a defined prevalence gap, effectively reducing the set of candidate *k*-mers by prioritizing *k*-mers with distinct prevalence differences between groups.

To exclude low-complexity regions, we only retain *k*-mers with Shannon entropy (Shannon 1948) ≥σ (default: σ=1.5). To mitigate sequencing artifacts, we further apply a homopolymer filter: *k*-mers are rejected if they contain a mononucleotide run exceeding either a relative length of 0.33×k or an absolute maximum of 15 bp. Additionally, *k*-mers composed of di- or trinucleotide repeats are removed.

### Unitig assembly and presence verification (Pass III & IV)

The set of retained *k*-mers, referred to as candidate *k*-mers, is assembled into unitigs using BCALM2 (Chikhi et al. 2016). Since overlapping *k*-mers may share sequence context, assembly reduces redundancy and reconstructs the genomic context required for valid association testing.

As no sample associations of the unitigs have been maintained in our workflow, we ascertain the presence of unitigs in the samples as follows: For each sample *s*, we compute all maximal exact matches (MEMs) of length ≥ℓ (default: ℓ=k+1) between the unitigs and the reads. A MEM can neither be extended on the left nor on the right by further matching positions. MEM computation relies on sampling *q*-mers from windows of ω=ℓ−q+1 consecutive *q*-mers (Marçais et al. 2019). With the default values of k=31 and q=19, this typically results in samples of ∼15 of all *q*-mers of a sequence.

A unitig is considered present in sample *s* if it satisfies the following: (i) there must be matches to at least $n_{\mathrm{reads}}$ distinct reads from $R_{s}$ (default: $n_{\mathrm{reads}}=3$); (ii) there must be at least one match covering *α* bases at the $3^{\prime }$-end and a match covering *α* bases at the $5^{\prime }$-end (default: α=12); (iii) at least $b_{\min,c}\;$ of the unitig is covered by matches, where, by default, $b_{\min,c}\;$ varies depending on the length of the unitig; and (iv) for long unitigs, there are at least two matches at the $3^{\prime }$-end and the $5^{\prime }$-end of distinct reads. This pass delivers, for each unitig, the number of samples per trait group in which it is present.

### Statistical association testing (Pass V)

Present unitigs undergo a final prevalence filtering with $T_{\min}=r_{\min}\times 0.2$ (yielding 10% in balanced sample sets) and $T_{\max}=1-T_{\min}$ to exclude ubiquitous core sequences and low-frequency noise based on the sample set’s minority ratio $r_{\min}$. Trait associations are quantified using Fisher’s Exact Test. To balance statistical rigor with sensitivity in small sample sets, the theoretical minimum achievable *p*-value is calculated ($p_{\min}$). Let *m* be the number of tested unitigs. If statistical power permits significant discovery under correction ($p_{\min}\leq \frac{\alpha }{m}$), the Benjamini-Hochberg procedure is applied (default α=0.05) (Benjamini and Hochberg 1995). Otherwise, the method reverts to a nominal significance threshold (default p≤0.01) to prevent too many false negatives in exploratory settings.

### Implementation

BiTUGA is implemented as a modular workflow orchestrated by a central shell script that integrates established external programs (KMC3 and BCALM2) and custom C++/Python programs. It is available on GitHub. For convenience, BiTUGA is compiled by executing make.

### Workflow execution

BiTUGA requires a directory of FASTQ files and a file mapping sample IDs to traits. Users can define resource limits (maximum threads and RAM). The complete analysis execution is performed from the repository root using:

./BiTUGA.sh \

--input-dir fastq_data/ \

--trait-info trait_info.tsv \

--outdir run_1/

--threads 12 \

--mem-gb 75

To avoid unnecessary computations due to parameter refinement in later passes, execution can be started from a specific pass (e.g. --start-pass 4) utilizing existing intermediate files. Upon completion, BiTUGA generates the following output directory tree:


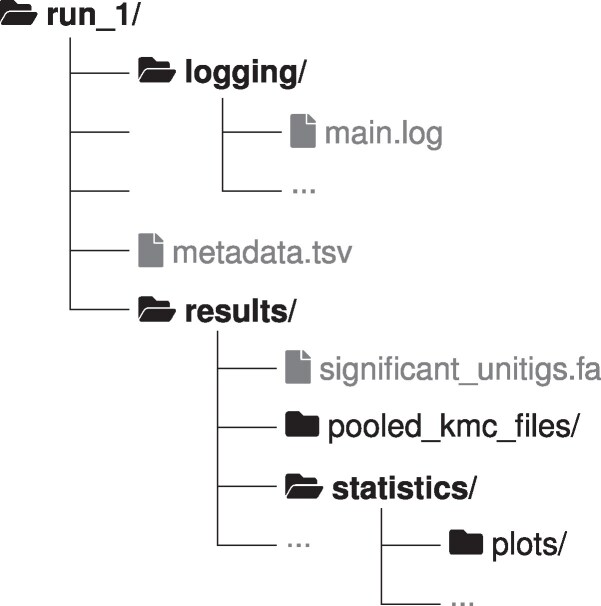


### Application to dioecious plants

We evaluated BiTUGA on two datasets representing distinct genomic complexities. For the angiosperm *Populus tremula* with a genome size ∼0.41Gbp, we analyzed a dataset from Wang et al. (2016) comprising 10 males and 10 females, utilizing the workflow’s default configuration. For the gymnosperm *Ginkgo biloba* with a genome size of 9.74Gbp (Liu et al. 2021), we analyzed a dataset from Liao et al. (2020) comprising 44 males and 44 females. Due to the large genome, we enabled the contrast filtering mode (--group-contrast) to improve the signal-to-noise ratio. To conduct an exploratory search for trait-associated genomic sequences and prioritize sensitivity for spatial clustering validation, we disabled the default FDR correction (--fdr none). Sample accession numbers, trait metadata are provided in Supplementary Data S1 and S2. Parameter settings and execution logs are provided in Supplementary Data S3.

For downstream validation, trait-associated unitigs were mapped to the chromosomal sequences of their respective male reference assemblies *P. tremula* W52-T195 (Müller et al. 2020) and *G. biloba* v2025 (Gu et al. 2022; Liu et al. 2021) using minimap2 (v2.30) (Li 2018) with default parameters. To visualize the genomic distribution of the unitigs, we calculated the fraction of covered bases per non-overlapping window.

## Results and discussion

BiTUGA processed both datasets with similar resources. For *G. biloba*, low resource use despite 5× more features than *P. tremula* was achieved by enabling --group-contrast (Table 1). This substantially reduced the candidate set after Pass II. By combining prevalence-based feature reduction with sample-wise processing, BiTUGA maintains a low memory footprint. Per-pass performance metrics are provided in Supplementary Data S3.

**Table 1.** Performance benchmarks under identical resource limits. Datasets were processed on a single compute node with 12 threads and a limit of 75 GB RAM. Input volume is reported as the total number of bases (Gbp; sum of read sequence lengths) and total size of gz-compressed FASTQ files.

Dataset	Genome size (Gbp)	Input (Gbp)	Input (gz, GB)	Input *k*-mers	Candidate *k*-mers	Runtime (h)	Peak RAM (GB)
*P. tremula*	0.41	344.71	319.95	$1.66\times 10^{9}$	$3.29\times 10^{8}$	∼14	∼70
*G. biloba*	9.74	747.74	329.67	$8.11\times 10^{9}$	$5.67\times 10^{6}$	∼25	∼70

**Fig. 2.**
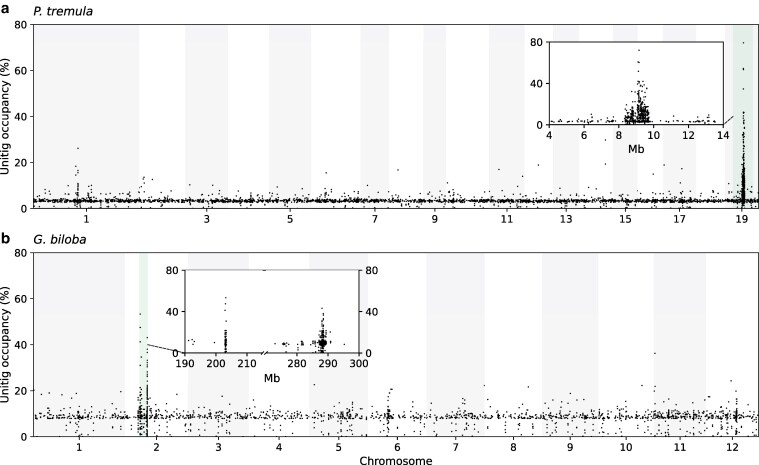
Genome-wide distribution of sex-associated unitigs. Unitigs were mapped to male reference assemblies. Occupancy denotes the fraction (%) of bases covered by significant unitigs within non-overlapping genomic windows. (a) *P. tremula* (Müller et al. 2020) (1,500 bp windows); inset highlights chromosome 19 (4–14 Mb). (b) *G. biloba* (Liu et al. 2021) (500 bp windows); inset highlights chromosome 2 (190–310 Mb).

BiTUGA identified 27,207 sex-associated unitigs in *P. tremula* and 47,357 in *G. biloba* at nominal p≤0.01. These unitigs were predominantly male-biased, consistent with the reported XY sex-determination systems (Müller et al. 2020; Gong and Filatov 2022). In *P. tremula*, the unitigs formed a dense cluster on chromosome 19 of a male mapping reference (Müller et al. 2020) with 96% of the top 50 occupancy windows within the 8.73–9.62 Mb interval (Fig. 2a; see Supplementary Data S4 for detailed mapping statistics). This region lies centrally within the broader signal peak (∼8.2−9.8 Mb) and is consistent with the ∼2 Mb SDR on chromosome 19 characterized previously (Kersten et al. 2014; Müller et al. 2020). In *G. biloba*, mapping to the male reference assembly (Liu et al. 2021) revealed unitig enrichment on chromosome 2, with 76% of the top 50 windows spanning 203.0–289.5 Mb (Fig. 2b). We observed local maxima at 200 Mb and 290 Mb. This aligns well with the SDR boundary (200.8 Mb) characterized by Gong and Filatov (2022). Unlike prior analyses based on female references that inferred the SDR from reduced male mapping (Gong and Filatov 2022; Liao et al. 2020), male-reference mapping used here yields a presence-based signal of sex-associated sequences. Within these intervals, the longest unitig reached 1,030 bp in *P. tremula* (∼9.17 Mb) and 92 bp in *G. biloba* (∼288.1 Mb), providing candidate sequences for genetic marker design, such as primer or probe development. Trait-associated unitigs are provided in Supplementary Data S3. An external support summary based on BLASTn searches against the NCBI nt database is provided in Supplementary Data S5 (Camacho et al. 2009; Sayers et al. 2025). In *P. tremula*, this analysis recovered 11 significant BLAST hits to ARR17, a well-established sex-related candidate locus in poplar SDR biology (Müller et al. 2020).

Unlike *P. tremula*, where unitigs reached up to 100 prevalence difference, the maximum in *G. biloba* corresponded to only ∼55 of male samples. Traditional *k*-mer approaches often rely on sex-specific sequences present in all samples of one trait group and absent in all samples of the other (Akagi et al. 2014; Carey et al. 2024) would likely overlook such a signal (Milewicz and Sawicki 2013; Palmer et al. 2019). On the other hand, BiTUGA successfully recovered the *G. biloba* SDR signal with its more flexible model.

This pattern may not reflect genuine genomic absence. Given the low mean sequencing depth (∼2×) in the *G. biloba* dataset (Liao et al. 2020), stochastic coverage dropouts and threshold-based presence calling can cause truly trait-specific sequences to be missed in individual samples (Lander and Waterman 1988). Thus, incomplete prevalence may reflect both limited detection power and biological heterogeneity in large, repetitive gymnosperm genomes, including limited differentiation of the underlying sex-determining systems (Charlesworth 2016; Walas et al. 2018). By replacing rigid presence/absence requirements with flexible prevalence thresholds, BiTUGA accommodates such variability and remains effective under these conditions. While evaluated in the present study for sex determination, the prevalence-based design of BiTUGA may also be applicable to other discrete binary traits, including bacterial antibiotic resistance or phage immunity (Jaillard et al. 2018; Kuang et al. 2026), qualitative disease resistance in large crop genomes (Jaegle et al. 2025), and structurally complex morphological traits such as polledness in livestock (Medugorac et al. 2012).

Notably, all results were obtained from raw reads, bypassing any read preprocessing. The ability to detect valid markers from raw data highlights the intrinsic resilience of the prevalence-based strategy. By requiring candidate *k*-mers to recur across multiple individuals, BiTUGA effectively filters *k*-mers derived from stochastic sequencing errors, adapters, and environmental contaminants that do not satisfy prevalence thresholds (Marchet et al. 2020). However, BiTUGA’s decisive computational advantage lies in the algorithmic design. In contrast, standard reference-free methods typically require constructing *k*-mer-by-sample matrices, a strategy that becomes disproportionate when scaling to billions of *k*-mers (Marchet et al. 2021; Rahman et al. 2018). For sequence association of discrete binary traits, maintaining such exhaustive data structures is not necessary. By bypassing global matrix construction and retaining only group-discriminative *k*-mers, BiTUGA eliminates this primary memory bottleneck.

## Data Availability

BiTUGA source code is available at https://github.com/JMittelbach/BiTUGA (MIT license). The software was tested on x86_64/Linux and Apple Silicon/macOS. *P. tremula* sequencing reads were retrieved from the NCBI SRA (BioProject PRJNA297202) (Wang et al. 2016). The reference assembly (W52-T195) was obtained from PlantGenIE, and phenotypic sex information for the selected individuals was obtained from the associated metadata (Müller et al. 2020). *G. biloba* sequencing reads were retrieved from the CNCB Genome Sequence Archive (accession CRA002039) (Liao et al. 2020), and the reference assembly (v2025) (Liu et al. 2021) was obtained from GinkgoDB (Gu et al. 2022). Supplementary materials are available at Figshare.
